# Supplementary material for: Functional Role of STIM-1 and Orai1 in Human Microvascular Aging
Source: Cells. 2022 Nov 18;11(22):3675. doi: 10.3390/cells11223675 (PMC9688234; doi:10.3390/cells11223675)
Supplement: Supplementary file 1 [file cells-11-03675-s001.zip › Suppl Fig S1 Cells 221105.pptx]

## Slide 1
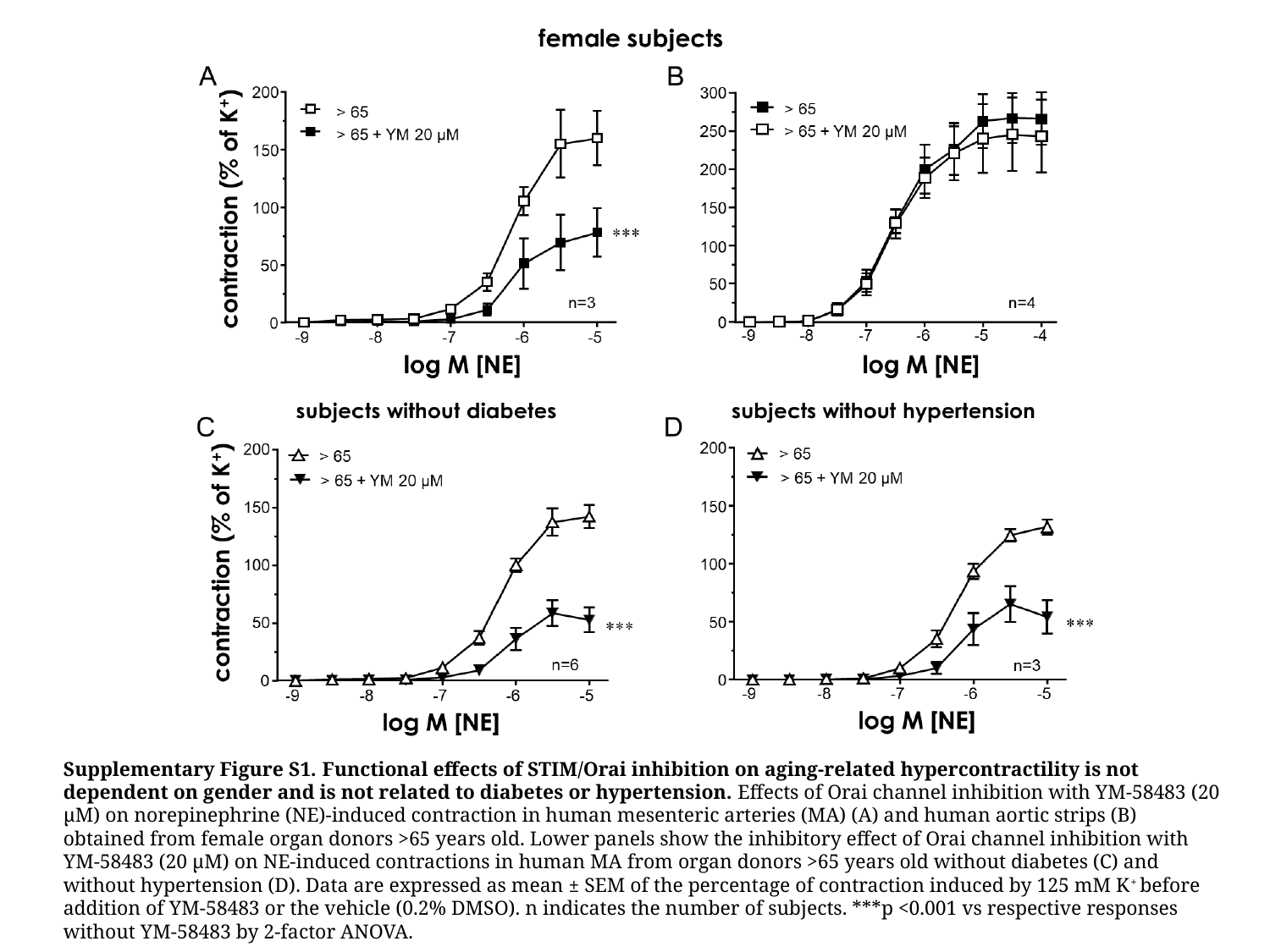

Supplementary Figure S1. Functional effects of STIM/Orai inhibition on aging-related hypercontractility is not dependent on gender and is not related to diabetes or hypertension. Effects of Orai channel inhibition with YM-58483 (20 µM) on norepinephrine (NE)-induced contraction in human mesenteric arteries (MA) (A) and human aortic strips (B) obtained from female organ donors >65 years old. Lower panels show the inhibitory effect of Orai channel inhibition with YM-58483 (20 µM) on NE-induced contractions in human MA from organ donors >65 years old without diabetes (C) and without hypertension (D). Data are expressed as mean ± SEM of the percentage of contraction induced by 125 mM K+ before addition of YM-58483 or the vehicle (0.2% DMSO). n indicates the number of subjects. ***p <0.001 vs respective responses without YM-58483 by 2-factor ANOVA.
